# Recent enhancement of central Pacific El Niño variability relative to last eight centuries

**DOI:** 10.1038/ncomms15386

**Published:** 2017-05-30

**Authors:** Yu Liu, Kim M. Cobb, Huiming Song, Qiang Li, Ching-Yao Li, Takeshi Nakatsuka, Zhisheng An, Weijian Zhou, Qiufang Cai, Jinbao Li, Steven W. Leavitt, Changfeng Sun, Ruochen Mei, Chuan-Chou Shen, Ming-Hsun Chan, Junyan Sun, Libin Yan, Ying Lei, Yongyong Ma, Xuxiang Li, Deliang Chen, Hans W. Linderholm

**Affiliations:** 1 The State Key Laboratory of Loess and Quaternary Geology, The Institute of Earth Environment, Chinese Academy of Sciences, Xi'an 710061, China; 2 Interdisciplinary Research Center of Earth Science Frontier (IRCESF) and Joint Center for Global Change Studies (JCGCS), Beijing Normal University, Beijing 100875, China; 3 School of Earth Sciences and Engineering, Nanjing University, Nanjing 210046, China; 4 School of Earth and Atmospheric Sciences, Georgia Institute of Technology, Atlanta, Georgia 30332, USA; 5 Department of Tourism and Leisure Management, Tung Fang Design Institute, Kaohsiung 82941, Taiwan; 6 Research Institute for Humanity and Nature, 457-4 Motoyama, Kamigamo, Kita-ku, Kyoto 603-8047, Japan; 7 Department of Geography, The University of Hong Kong, Pokfulam Road 999077, Hong Kong; 8 The Laboratory of Tree-Ring Research, The University of Arizona, Tucson, Arizona 85721, USA; 9 High-Precision Mass Spectrometry and Environment Change Laboratory (HISPEC), Department of Geosciences, National Taiwan University, Taipei 10617, Taiwan; 10 Department of Forestry and Natural Resources, National Chiayi University, Chiayi 60004, Taiwan; 11 School of Human Settlements and Civil Engineering, Xi'an Jiaotong University, Xi'an 710049, China; 12 Regional Climate Group, Department of Earth Sciences, University of Gothenburg, S-405 30 Gothenburg, Sweden

## Abstract

The far-reaching impacts of central Pacific El Niño events on global climate differ appreciably from those associated with eastern Pacific El Niño events. Central Pacific El Niño events may become more frequent in coming decades as atmospheric greenhouse gas concentrations rise, but the instrumental record of central Pacific sea-surface temperatures is too short to detect potential trends. Here we present an annually resolved reconstruction of NIÑO4 sea-surface temperature, located in the central equatorial Pacific, based on oxygen isotopic time series from Taiwan tree cellulose that span from 1190 AD to 2007 AD. Our reconstruction indicates that relatively warm Niño4 sea-surface temperature values over the late twentieth century are accompanied by higher levels of interannual variability than observed in other intervals of the 818-year-long reconstruction. Our results imply that anthropogenic greenhouse forcing may be driving an increase in central Pacific El Niño-Southern Oscillation variability and/or its hydrological impacts, consistent with recent modelling studies.

## Abstract (web summary)

El Niño events in the Central Pacific may be changing due to climate change, but long records to support this are lacking. Here, the authors present sea surface temperature reconstructions from tree cellulose for the last 800 years which suggest the variability of Central Pacific El Niño events has increased.

Sea-surface temperature (SST) variations in the tropical Pacific and associated changes in global atmospheric circulation dominate global climate variability on interannual timescales. Recent studies distinguish between canonical El Niño events, with warming located in the central to eastern tropical Pacific, and central Pacific (CP) El Niño events, wherein warming is confined to the central tropical Pacific^1^,^2^,^3^. Both CP and the East Pacific El Niño-Southern Oscillation (ENSO) events have widespread impacts on global climate^4^,^5^,^6^,^7^, including influences on western North American drought^8^, East Asian monsoons^9^ and hurricane properties^6^. Climate model simulations suggest that CP ENSO variability may increase under greenhouse forcing^1^,^10^,^11^, but instrumental records of tropical Pacific SSTs are too short to provide robust constraints on recent trends in ENSO variability.

Instrumental and modelling studies use indices of large-scale SST variability in the central to western tropical Pacific—the NIÑO3.4 and NIÑO4 indices, respectively—to quantify CP ENSO variability through time^1^,^2^. While there are several high-resolution, multi-century reconstructions of NIÑO3.4 SST^12^,^13^,^14^,^15^, only two such reconstructions of NIÑO4 SST exist—one derived from an ice core in Peru^16^ and another based on tree-rings in southwest America^17^. Additional multi-century reconstructions of CP SSTs are required to improve quantification of the response of CP ENSO variability to both natural and anthropogenic climate forcings.

Here we present an 818-year-long, annually resolved record of tree-ring cellulosic oxygen isotopic (δ^18^O) composition from Taiwan, a region where CP ENSO-related changes in atmospheric circulation and hydroclimate are large^9^,^18^,^19^. Tree-ring cellulosic δ^18^O is a well-established proxy sensitive to large-scale hydrological conditions^20^. Our results show that the warm phases of our reconstruction correspond to strong El Niño years. The annual variation and variance of NIÑO4 SST are relatively high during the late twentieth century likely due to anthropogenic global warming.

## Results

### Taiwan tree-ring δ^18^O chronology

**Figure 1:**
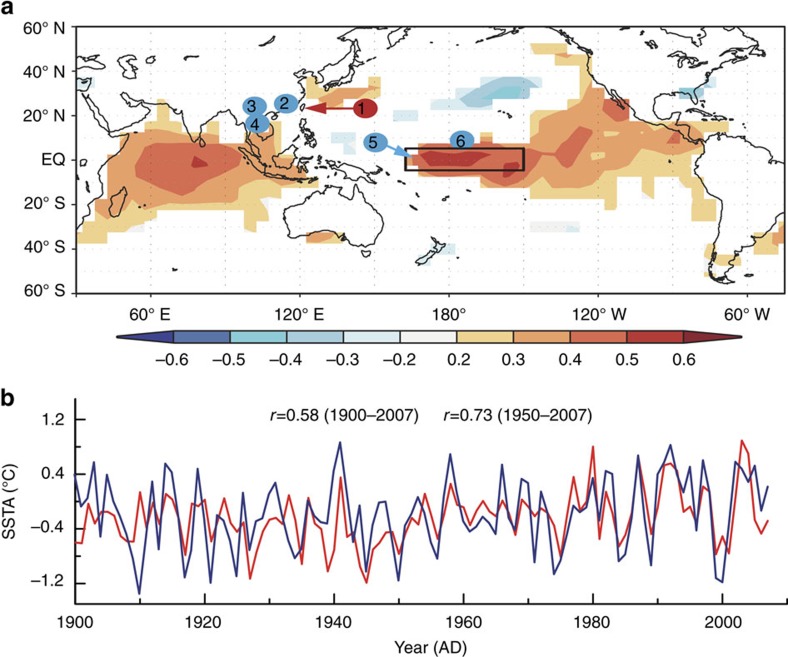
Map and time series showing the relationship of Taiwan tree-ring δ^18^O with regional sea-surface temperature (SST). (**a**) Map of the composite Taiwan tree-ring δ^18^O record regressed upon global SSTs^52^ (https://www.esrl.noaa.gov/psd/data/gridded/data.kaplan_sst.html) from 1900 to 2007 AD. Colours define areas of statistically significant correlations (*P*<0.05). The black rectangle denotes the NIÑO4 region. The shaded numbers denote the locations of proxy records mentioned in the text: 1—Taiwan tree-ring ^18^O; 2—Fujian, China tree-ring δ^18^O (ref. 27, 25° 59′ N, 106° 26′ E, 1901–2004 AD); 3—Mu Cang Chai, Laos tree-ring δ^18^O (ref. 28, 21° 40′ N, 104° 06′ E, 1705–2005 AD); 4—PhuLeuy Mountain, Vietnam tree-ring δ^18^O (ref. 29, 20° 17′ N, 103° 55′ E, 1688–2002 AD); 5—Maiana coral δ^18^O (ref. 33, 1° N, 173° E, 1840–1995 AD); 6—Palmyra coral δ^18^O (ref. 12, 6° N, 162° W, 1635–1703 AD, 1886–1998 AD). (**b**) Comparison of the annually resolved SST anomaly (SSTA) between Taiwan tree-ring δ^18^O-based NIÑO4 index (red) and the Kaplan instrumental NIÑO4 index^21^ averaged from March to May (blue) of each year (*P*<0.05). Note that the significance of all correlations reported in this study have been assessed using effective degrees of freedom that account for autocorrelation in the time series^27^. Map of **a** was created by http://climexp.knmi.nl/corfield.cgi.

In total, 50 *Chamaecyparis formosensis* Matsum tree-ring cores from 29 individual trees were collected from Mt. Daxue, Taiwan (∼24° N, 121° E) at an elevation of 2,000–2,200 m above sea level (Fig. 1a). After cross-dating the ring-width time series across every core (Supplementary Table 1), 16 cores were selected for cellulose δ^18^O analysis following standard protocols (Methods).

**Figure 2:**
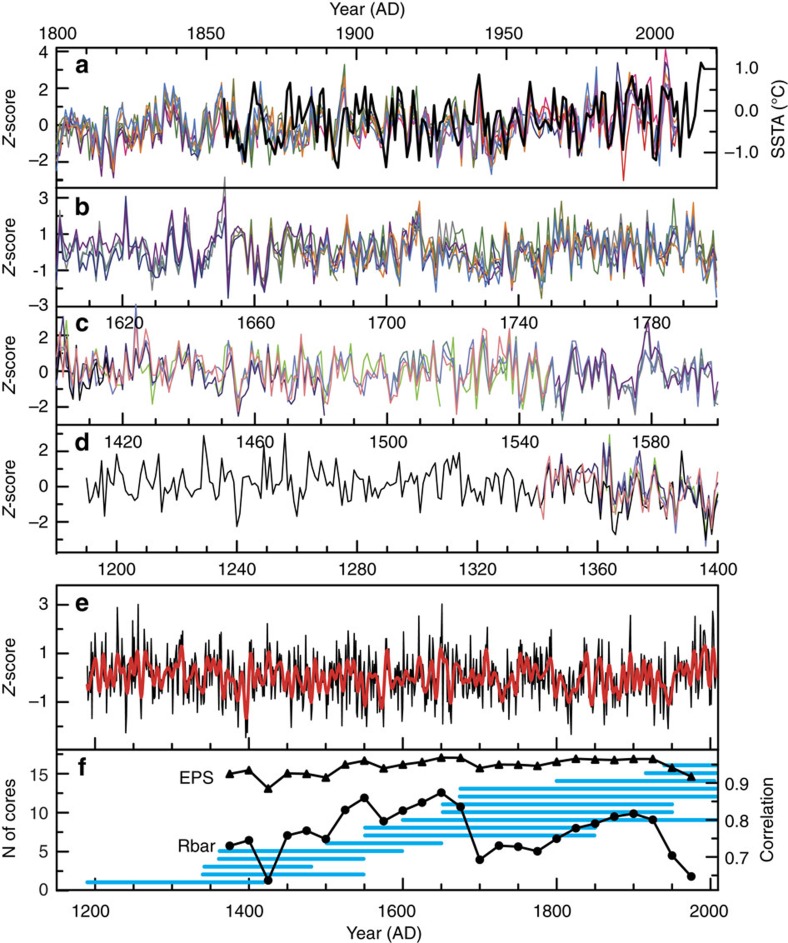
Taiwan tree-ring δ^18^O records and associated reconstruction metrics. (**a**–**d**) Replicate annually resolved δ^18^O time series from 16 individual trees from Mt. Daxue, Taiwan (thin coloured lines) plotted with the Kaplan NIÑO4 sea-surface temperature index averaged from March to May of each year (thick black line, 1856–2015 AD). (**e**) Normalized composite tree-ring δ^18^O record (black line), plotted with an 8-year low-pass filter (red line). (**f**) The expressed population signal (EPS^33^,^34^) and Rbar^33^,^34^ (the average correlation between the δ^18^O series for each year over the sequential time periods) statistics of the δ^18^O reconstruction (Methods), and number of cores available through the reconstruction interval. All tree-ring δ^18^O series in **a**–**e** were normalized (*Z*-score). SSTA, SST anomaly.

To avoid potential δ^18^O artefacts associated with juvenile isotope effects^21^, we excluded the first 20 years (rings)^21^,^22^,^23^,^24^,^25^ from those cores with no visible rot in the pith, following standard procedures. The resulting 16 annually resolved time series contain overlapping segments of 67–408 years in length (Fig. 2a–d,f), and have significant common variability where they do overlap (*r*=0.51–0.89, *P*<0.001; Supplementary Table 2). In generating a composite tree-ring δ^18^O record from this ensemble, we arithmetically averaged the 16 individual δ^18^O time series into a single δ^18^O time series spanning 1190–2007 AD (Fig. 2e). Aside from the high degree of between-sample reproducibility of cellulosic δ^18^O, a variety of standard statistical metrics confirms that the composite Taiwan tree-ring δ^18^O time series is robust (Fig. 2f; Methods).

### Climate signals of Taiwan tree-ring composite δ^18^O record

**Figure 3:**
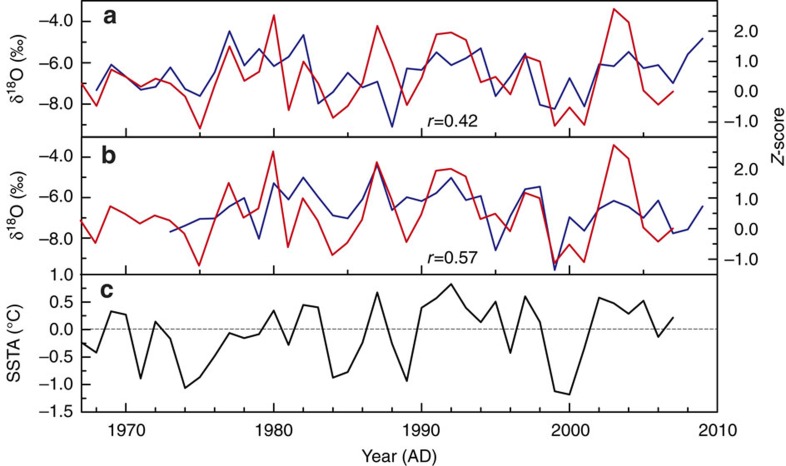
Comparison between the Taiwan tree-ring δ^18^O (red) and the precipitation δ^18^O (blue) in the adjacent western Pacific obtained from the Global Network of Isotopes in Precipitation. (**a**) *r*=0.42 with Bangkok precipitation δ^18^O (*n*=40, *P*<0.0001). (**b**) *r*=0.57 with Hong Kong (*n*=35, *P*<0.0001). These plots suggest that the Taiwan tree-ring δ^18^O reflects large-scale regional hydrological signals. (**c**) NIÑO4 sea-surface temperature anomaly time series (http://www.cpc.ncep.noaa.gov/products/analysis_monitoring/ensostuff/ensoyears.shtml). The horizontal line denotes 0 °C value. SSTA, SST anomaly.

The Taiwan tree-ring composite δ^18^O record is a sensitive indicator of regional hydroclimate, as indicated by significant correlations with regional precipitation δ^18^O time series (Methods; Fig. 3). At the local scale, the record is significantly correlated to temperature, precipitation and relative humidity (RH), such that higher tree δ^18^O values reflect warm, dry conditions (Supplementary Fig. 1a). Indeed, the Taiwan tree δ^18^O record is significantly correlated to the mean value of May–September Palmer Drought Severity Index^26^ (at the grid point 24.75° N, 121.75° E, *r*=−0.45, *P*<0.0001), consistent with previous studies of tree δ^18^O time series from southeast Asia^27^,^28^,^29^. Correlation analysis also showed significant relationship between Taiwan Palmer Drought Severity Index and regional precipitation (Supplementary Fig. 2).

### Relationship between NIÑO4 SST and tree-ring δ^18^O

**Figure 4:**
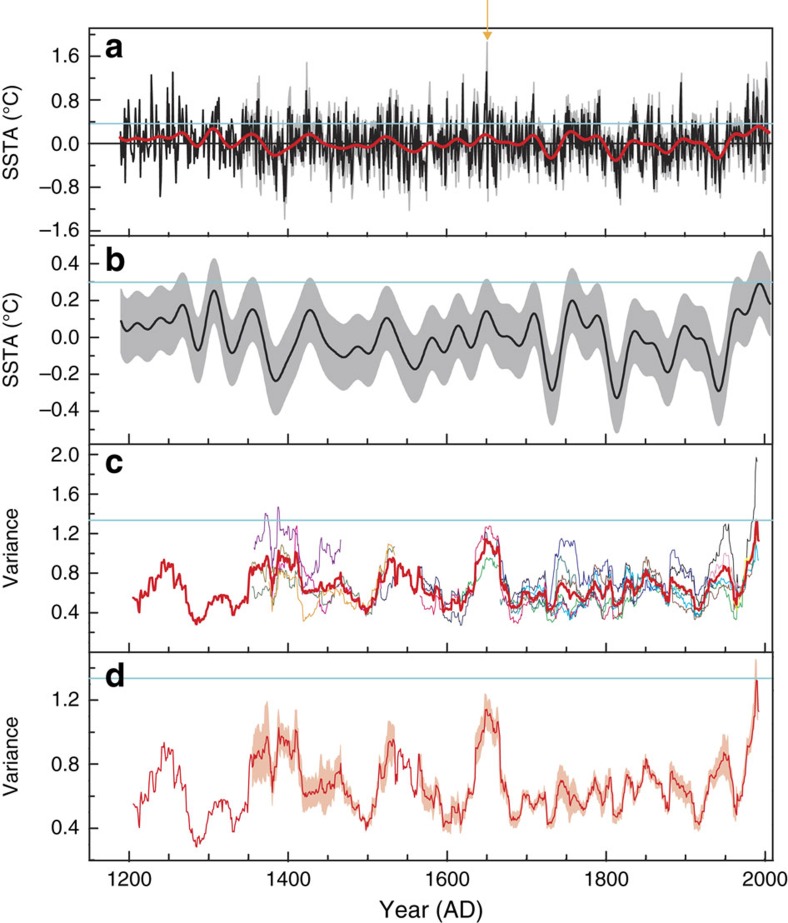
Composite Taiwan tree-ring δ^18^O-based reconstruction of NIÑO4 sea-surface temperature (SST) anomaly from 1190 to 2007 AD. (**a**) Plot of composite tree δ^18^O-based NIÑO4 SST anomalies (SSTAs) averaged over March–May for each year, calculated with respect to the mean of observed SSTs during the 1950–2007 AD period (black horizontal line), plotted with 31-year low-passed version of the data (red line), and the blue horizontal line indicates the highest 31-year low-passed SST value of the time series. The grey area denotes ±2*σ* error bars, based on the statistical reconstruction across overlapping tree δ^18^O series^53^. An anomalously high reconstructed SST value in 1651 AD is indicated by an orange arrow. (**b**) Thirty-one-year low-passed value of the composite Taiwan tree δ^18^O series shown in **a**. The grey area denotes ±2*σ* smoothed error bars^54^. The blue horizontal line indicates the highest 31-year low-passed SST value of the time series^41^ (Methods). (**c**) Time series of 31-year running variance of internnual-scale variability (isolated with a 2–7-year band-pass filter) corresponding to each of the 16 individual raw tree-ring δ^18^O time series (thin coloured lines) and the average of these running variance time series (thick red line, for periods where they overlap). (**d**) The red line is the same red line in **c** and the pink area denotes ±1*σ* of the mean. The horizontal blue lines both in **c**,**d** indicate the highest 31-year averaged interannual variance value of the time series, centred on 1992 AD.

The new Taiwan tree δ^18^O record is significantly correlated with NIÑO4 SST, located in the heart of the CP ENSO region (Fig. 1; Supplementary Fig. 3). Correlations are highest after 1950, when the quality of tropical Pacific SST data is highest^30^ (Supplementary Fig. 4a). We also computed correlations between our Taiwan tree-ring δ^18^O series and other proxy-based reconstructions of NIÑO3.4 and NIÑO4 SST, and find that proxy–proxy correlations are higher in the early 20th century than the corresponding proxy-SST correlations (Supplementary Table 3). Our analyses suggest that the quality of instrumental data during the early 20th century may be somewhat reduced relative to the late twentieth century. Given that Taiwan δ^18^O-NIÑO4 SST correlations are highest during March–May (Supplementary Fig. 1b; Supplementary Table 4), we used the tree δ^18^O record to reconstruct the March–May NIÑO4 SST from 1190 to 2007 AD (Fig. 4a). Calibration and verification metrics (Methods; Supplementary Fig. 4b; Supplementary Table 5) confirm that our Taiwan tree δ^18^O-based reconstruction of NIÑO4 SST is robust throughout its length.

**Figure 5:**
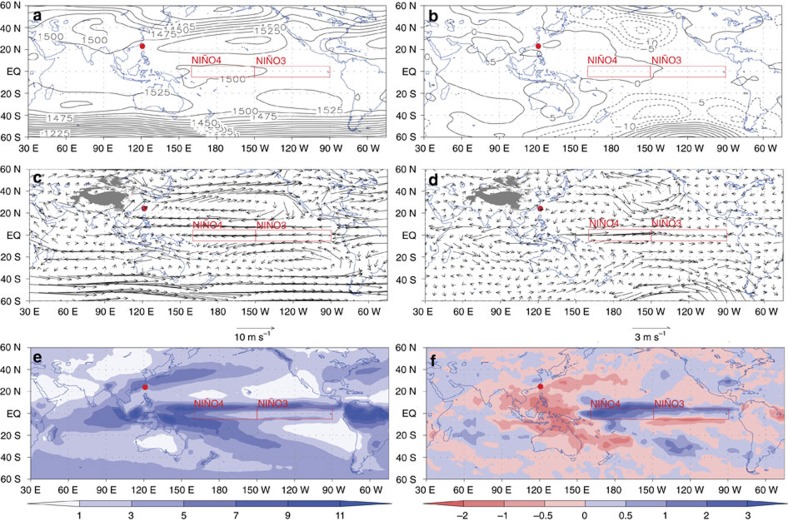
Climate variability in Taiwan related to central Pacific sea-surface temperature. Long-term (1981–2010) mean geopotential height filed (**a**, unit: geopotential metres, gpm) and wind field (**c**, unit: m s^−1^) at the 850 hPa pressure level retrieved by the National Centers for Environmental Prediction/the National Center for Atmospheric Research reanalysis data^32^, and precipitation field (**e**, unit: mm per day) retrieved by global precipitation climatology project data^55^ in the reconstruction season (March–April–May), and the composite anomaly fields of 850 hPa geopotential height (**b**), 850 hPa wind (**d**) and precipitation (**f**) in eight selected central Pacific El Nino years (1987, 1991, 1992, 1993, 1995, 1997, 2002 and 2005). Red dots are the location of Taiwan. This map was created using the software OpenGrADS Version 2.0.a8.

The positive correlation between Taiwan tree δ^18^O and the boreal spring NIÑO3.4 index reflects drier conditions across the western Pacific region during El Niño events. Time series of rainfall δ^18^O from the Global Network of Isotopes in Precipitation database confirm that during CP El Niño events, the δ^18^O of rainfall increases across the western Pacific as the locus of large-scale convection shifts away from the maritime continent to the CP (Methods; Fig. 3). These results suggest that the Taiwan tree δ^18^O record tracks local changes in rainfall δ^18^O, in line with findings from forward modelling studies of tree cellulose δ^18^O variability^20^. Previous studies have documented that local rainfall δ^18^O is more sensitive to ENSO variability than local rainfall amount in the western tropical Pacific^27^,^28^,^29^,^31^, given the appreciable spatial and temporal averaging inherent in the rainfall δ^18^O variations. Further support for the CP El Niño-Taiwan hydrological link comes from positive 850 hPa geopotential height anomalies in March–May in South Asia and the western Pacific during CP El Niño events^32^ (Fig. 5). The associated large-scale anticyclonic flow during CP El Niño extremes causes subsidence and the weakening of the prevailing southwest winds over Taiwan, both of which contribute to precipitation decreases throughout the region of interest. Collectively, these analyses provide a dynamical context for the observed correlations between the Taiwan tree δ^18^O record and the NIÑO4 SST index—the target of our reconstruction.

### Characteristics of the reconstructed NIÑO4 SST

The Taiwan tree δ^18^O-based SST reconstruction contains a rich spectrum of variability spanning interannual to centennial timescales (Fig. 4; Supplementary Figs 5 and 6), similar to other multi-century reconstructions of tropical Pacific SSTs^14^. Several individual years stand out as exceptionally warm, allowing for the potential identification of strong El Niño years over the last millennium. On multi-decadal to century timescales, reconstructed SST values during the late 20th century are significantly higher than during any previous interval (Fig. 4d; Supplementary Table 6), consistent with anthropogenic warming of the NIÑO4 region. Indeed, anomalous late twentieth century warming in the central tropical Pacific is also inferred from coral δ^18^O time series from Maiana^33^ and Palmyra^12^ (Supplementary Fig. 7), as well as from a tree-ring-based reconstruction of NIÑO3.4 (ref. 14; Supplementary Fig. 8), all of which exhibit significant correlations with the Taiwan tree δ^18^O record over their periods of overlap (Supplementary Table 7).

Prominent interannual variability dominates the Taiwan tree δ^18^O record (Supplementary Figs 5 and 6), with a spectral signature similar to that observed in instrumental time series of the ENSO phenomenon. Before the twentieth century, the largest interannual excursions occur during the early to mid-seventeenth century, in line with previous observations of enhanced ENSO activity during this time^12^, possibly related to enhanced volcanic activity^34^. Indeed, the highest single tree-ring δ^18^O value of the entire reconstruction corresponds to 1651 AD, and may be linked to an exceptionally large El Niño event documented in historical records from the Paraná River region in South America^35^ as well as in coral δ^18^O records from the central tropical Pacific^12^,^33^ (Fig. 4a; Supplementary Fig. 7a,b). Interannual SST variance reaches a relative maximum during the late twentieth century (Fig. 4d), although appreciable spread in the individual tree-ring series precludes a finding of significance at the 95% confidence level. Taken at face value, our results provide empirical support for model projections of increased CP ENSO activity under continued anthropogenic climate change^2^,^10^. As such, the fact that the record-breaking 2015/2016 El Niño event was characterized by maximum warming in the CP, as opposed to the eastern Pacific, is consistent with a growing body of observational and modelling evidence for a prevalence of CP ENSO under greenhouse forcing.

## Discussion

Taken together, our results suggest that anthropogenic climate change has had a profound effect on SSTs in the CP, whereby anomalous warming over the last decades is accompanied by an increase in interannual variance. NIÑO4 SST values over the last two decades are likely higher than natural variations over the last 818 years, owing to a combination of relatively high CP ENSO activity and a late 20th century warming trend. In light of our results, it seems plausible that the dominance of CP ENSO extremes in the first two decades of the twenty-first century may continue, albeit with some important caveats. First, the global climate impacts of future CP ENSO extremes will critically depend on the evolution of the mean climate state in the tropical Pacific^36^,^37^, which itself is poorly constrained at present. Second, the new Taiwan tree δ^18^O record is the newest addition to growing archive of high-resolution paleo-data sets that can be used to probe the sensitivity of tropical Pacific climate to a variety of external climatic forcings over the recent past. One such example comes from the early- to mid-Holocene, when some models and data suggest that processional insolation forcing may have driven a shift towards greater CP ENSO activity and less East Pacific ENSO activity^37^. Should the dominance of CP ENSO extremes continue in the coming decades, investigations of the causes, and consequences, of any past shifts towards CP ENSO may provide some clues about future tropical Pacific climate trends and their global impacts.

## Methods

### Sample selection and cellulose extraction

Fifty tree-ring cores from 29 *Chamaecyparis formosensis* Matsum trees were collected from Taiwan in 2008. According to the standard dendrochronologial techniques, samples were polished and cross-dated. The quality of cross-dating was validated by program COFECHA^38^. Each individual tree-ring was identified as a calendar year. After accurate cross-dating, we selected 16 cores from each of 16 individual trees to carry out stable oxygen isotope analysis on the principle of ensuring at least 4 cores were present over the whole record.

The tree-ring cellulose was extracted as follows: materials of whole annual ring were separated and sliced into thin sections by a razor under microscope; the thin sections cut with a razor knife allow the chemical processing to proceed completely and rapidly; the sliced samples were chemically treated by acetone, a mixture of toluene and ethanol, acidified sodium hydrochlorite and 17.5% solution of sodium hydroxide in successive steps^39^,^40^; the cellulose of annual rings was transferred to a small bottle and homogenized with an ultrasonic cell crusher (JY92-2D, Ningbo Scientz Biotechnology Co., Ningbo, China); and the cellulose samples were dried overnight.

### Stable isotopic analysis

We loaded each 130–170 μg homogenized cellulose sample into a silver capsule. Each silver capsule with an annual sample was sealed and packed. The samples were converted to CO at 1,350 °C using pyrolysis-type elemental analyser (TC/EA, Thermo Fisher, Germany) interfaced to an isotope ratio mass spectrometer (Delta V Advantage, Thermo Fisher, Germany). The ^18^O/^16^O ratio was expressed in delta (δ^18^O) notation with reference to a standard material for which the isotopic ratio is known (equation (1)). The δ^18^O was determined from the following equation:


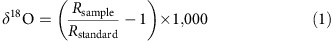


where *R*_sample_ and *R*_standard_ are the ^18^O/^16^O ratios for the sample and standard cellulose, respectively. Values of δ^18^O were reported with respect to the Vienna Standard Mean Ocean Water. The analytical reproducibility by analysing Merck cellulose (Merck KGaA, Darmstadt, Germany) was ±0.2‰.

### Tree-ring δ^18^O chronology development

We used a Numerical Mix Method^41^ to establish an accurate and reliable Taiwan tree-ring δ^18^O chronology. The idea behind Numerical Mix Method is that several individual δ^18^O series are measured first, and then the mean values are calculated using an arithmetic average to produce a single isotope chronology. This method treats the stable isotope series such as a tree-ring index, akin to ring width. It follows the standard procedure of tree-ring width chronology development by measuring individual tree-rings and creating a mean site value^42^,^43^,^44^.

The individual δ^18^O series were combined into a single chronology by computing arithmetical mean. There is no reason to suspect that the Taiwan tree-ring δ^18^O series should not preserve low-frequency climate signals. EPS, the expressed population signal^45^,^46^, is used to evaluate the agreement between the δ^18^O series (or the common variance relative to the total variance). Generally, that an EPS value is >0.85 is considered to be an acceptable threshold for a reliable chronology^45^,^46^. The Rbar^45^ parameter indicates the average correlation between the δ^18^O series for each year over the sequential time periods. In this study, EPS and Rbar were calculated for Taiwan tree δ^18^O chronology by using a 50-year window that lags by 25 years.

### Tree-ring δ^18^O responses to local climate parameters

The Yilan meteorological station (1936–2007 AD, 24° 46′ N, 121° 45′ E; 8 m above sea level) is the closest meteorological station to the sampling site with sufficiently complete records for climate response analysis. Thus, the observed precipitation, temperature and RH records from Yilan were used to identify the tree-ring δ^18^O climatic response. Considering the possibility that the climate of the current year not only affects tree growth in the current year but also in subsequent years^47^, we incorporated meteorological data from November of the prior year to October of the current year into our model. The monthly mean temperature, mean precipitation and mean RH of Yilan station are shown in Supplementary Fig. 9.

### Tree-ring δ^18^O responses to precipitation δ^18^O time series

Generally, the amount of precipitation shows a negative correlation with δ^18^O of precipitation at lower latitudes, which is referred as the ‘Amount Effect'^48^. Decreasing amount of precipitation in the western Pacific region during El Niño enriches the ratio of ^18^O/^16^O in precipitation. Tree physiology has demonstrated that δ^18^O of tree-rings was positively correlated with δ^18^O of precipitation and negatively correlated with RH^18^. However, there was no strong correlation between the local RH and the tree-ring δ^18^O (*r*=−0.21, *n*=72, *P*>0.01) at Taiwan sampling site, which implies that the δ^18^O of precipitation was the most important factor for determining the tree-ring δ^18^O value.

On the other hand, the Taiwan tree-ring δ^18^O series was significantly correlated with the precipitation δ^18^O records from the adjacent western Pacific region obtained from Global Network of Isotopes in Precipitation: *r* was 0.42 with Bangkok precipitation δ^18^O (*n*=40, *P*<0.0001), 0.68 with Kunming (*n*=16, *P*<0.001) and 0.57 with Hong Kong (*n*=35, *P*<0.0001; Fig. 3). Therefore, enriched ^18^O/^16^O of precipitation resulted in high δ^18^O value of tree-rings in Taiwan (including the western Pacific) during El Niño events and vice versa. It suggests that the Taiwan tree-ring δ^18^O contained large-scale precipitation δ^18^O signals in the northwestern Pacific sector.

### Split calibration-verification method

Analysis revealed that the Taiwan tree-ring δ^18^O is highly correlated with NIÑO4 SST from March to May during 1950–2007 (SST_MAM_, Supplementary Fig. 1b). A transfer function was then designed to reconstruct the central Pacific NIÑO4 SST using Taiwan tree-ring δ^18^O:


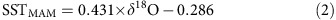


(*n*=58, *r*=0.734, *R*^2^=0.539, *R*^2^_adj_=0.531, *F*=65.546, *P*<0.0001, *D/W*=1.82).

As shown in Fig. 1b, the reconstructed SST matched the observed Kaplan NIÑO4 SST pretty well. The *r* was 0.64 after first difference (1951–2007, *P*<0.0001, Supplementary Fig. 4b), indicating their significant and stable relationships in high frequency.

The stability and reliability of the regression equation were evaluated using the split calibration-verification method^45^,^49^. It was performed by calibrating the NIÑO4 SST data from a subperiod (the data set was divided into two parts, 1950–1979 and 1978–2007) and verifying the reconstruction using the remaining data. The results were evaluated by the correlation coefficient (*r*), the sign test (ST), the reduction of error test (RE), the coefficient of efficiency (CE) and the product means test (*t*) during the verification period. Generally, RE and CE values greater than zero indicate a rigorous model skill^49^. Larger values of the RE and CE indicate better results. Moreover, the values of CE are more rigorous and are typically lower than those of RE (Supplementary Table 5).

As shown in Figs 1b and 2a and Supplementary Fig. 4b, the reconstructed SST matched the observed Kaplan NIÑO4 SST pretty well.

### The effective number of degree of freedom estimation

Since there are autocorrelations in the data used in this study and their corresponding number of degree of freedom is reduced, the effective number of degree of freedom (EDOF) is estimated to test the significance level of correlations for each pair of time series. In this paper, we used the method described by Bretherton *et al*.^50^ for estimating EDOF. The EDOF is estimated by:


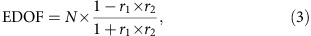


where *N* denotes the length of the time series, and *r*_1_ and *r*_2_ refer to the lag-one autocorrelation of each series, respectively.

### Smoothing method

The data at the ends of the time series before smoothing were padded by using the mean value of the remaining data. When there were 30 remaining data, their mean value was used as the 31th data. So that there were enough 31 data used to calculate the 31-year low-pass filter value. And, this process was repeated until the last year^51^.

### Data availability

Data that have contributed to the reported results are available from the corresponding author on request.

## Additional information

**How to cite this article:** Liu, Y. *et al*. Recent enhancement of central Pacific El Niño variability relative to last eight centuries. *Nat. Commun.* **8,** 15386 doi: 10.1038/ncomms15386 (2017).

**Publisher's note:** Springer Nature remains neutral with regard to jurisdictional claims in published maps and institutional affiliations.

## Supplementary Material

Supplementary Information Supplementary Figures, Supplementary Tables and Supplementary References
