# Supplementary material for: Recent enhancement of central Pacific El Niño variability relative to last eight centuries
Source: Nat Commun. 2017 May 30;8:15386. doi: 10.1038/ncomms15386 (PMC5459944; doi:10.1038/ncomms15386)
Supplement: Supplementary Information — Supplementary Figures, Supplementary Tables and Supplementary References [file ncomms15386-s1.pdf]

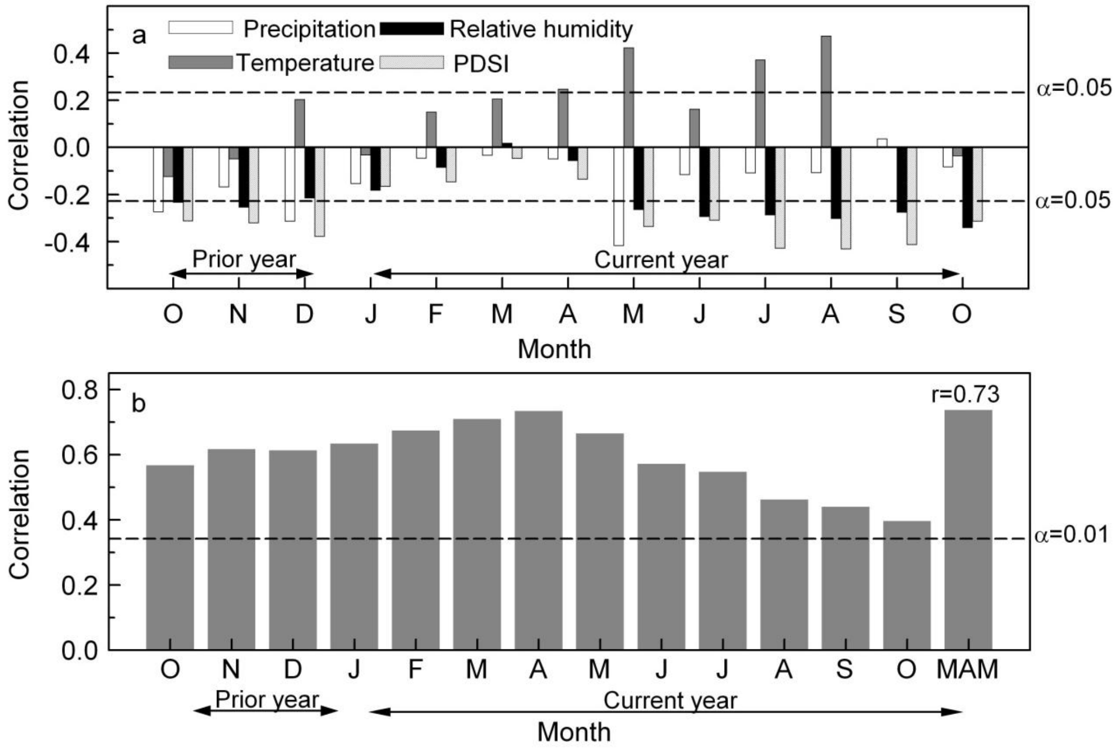

Supplementary Figure 1 | Correlations between Taiwan tree-ring δ^{18}O and instrumental climate datasets. a, Correlations between Taiwan tree-ring δ^{18}O and temperature, precipitation and relative humidity from Yilan station, and PDSI [1] from the nearest grid point [2] (CRU scPDSI 3.21, 24.75°N, 121.75°E, 1951–2007) to our sampling site. These plots show that the Taiwan tree-ring δ^{18}O chronology is positively correlated with temperature and negatively correlated with precipitation during the growing season (April–September), consistent with our interpretation that high tree-ring δ^{18}O values indicate dry, hot conditions. Indeed, the PDSI from May to October is significantly correlated with the Taiwan tree-ring δ^{18}O chronology with r=–0.44 (p<0.0001, 1936–2005). This interpretation is supported by tree-ring δ^{18}O studies in Laos, Vietnam, and Fujian, China [3–5]. **b**, Correlation function analyses over the period 1950–2007 between the annually-resolved Taiwan tree-ring δ^{18}O and Kaplan NIÑO4 SST for each month, as well as the March-May average (MAM, far right column) which is our chosen reconstruction target.

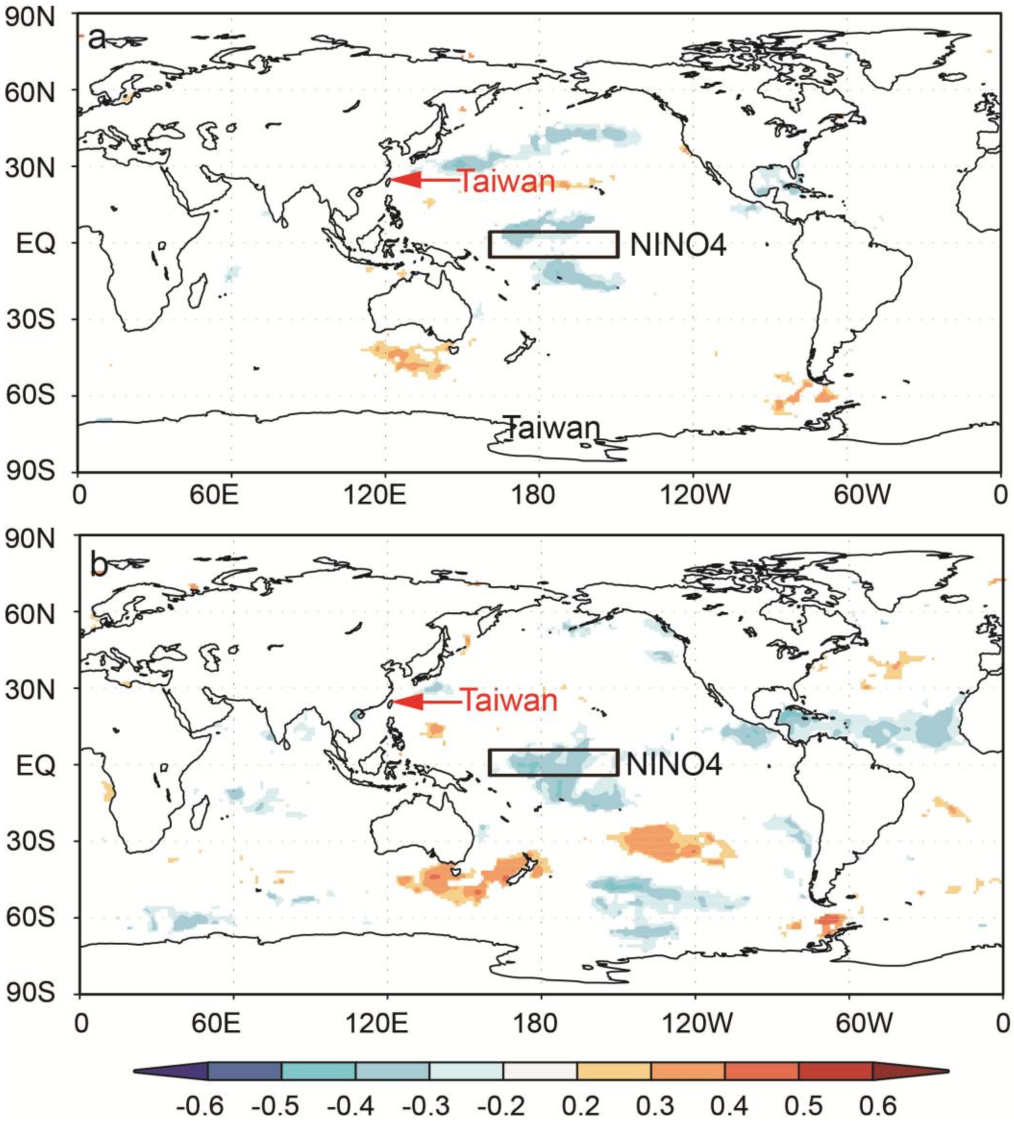

Supplementary Figure 2 | Spatial correlation patterns between Hadley SSTs [6] and (a) Total precipitation from March to May from Yilan (1950–2007) using CRU TS3.23 [7] and (b) Averaged March to May PDSI [1] of Yilan during period 1950–2007 [8] (*p*<10%) (CRU scPDSI 3.21, 24.75 °N, 121.75 °E). These indicate that NIÑO4 SST indeed influences the Taiwan precipitation and PDSI. The black rectangle denotes the NIÑO4 region. This map was created by http://climexp.knmi.nl/corfield.cgi.

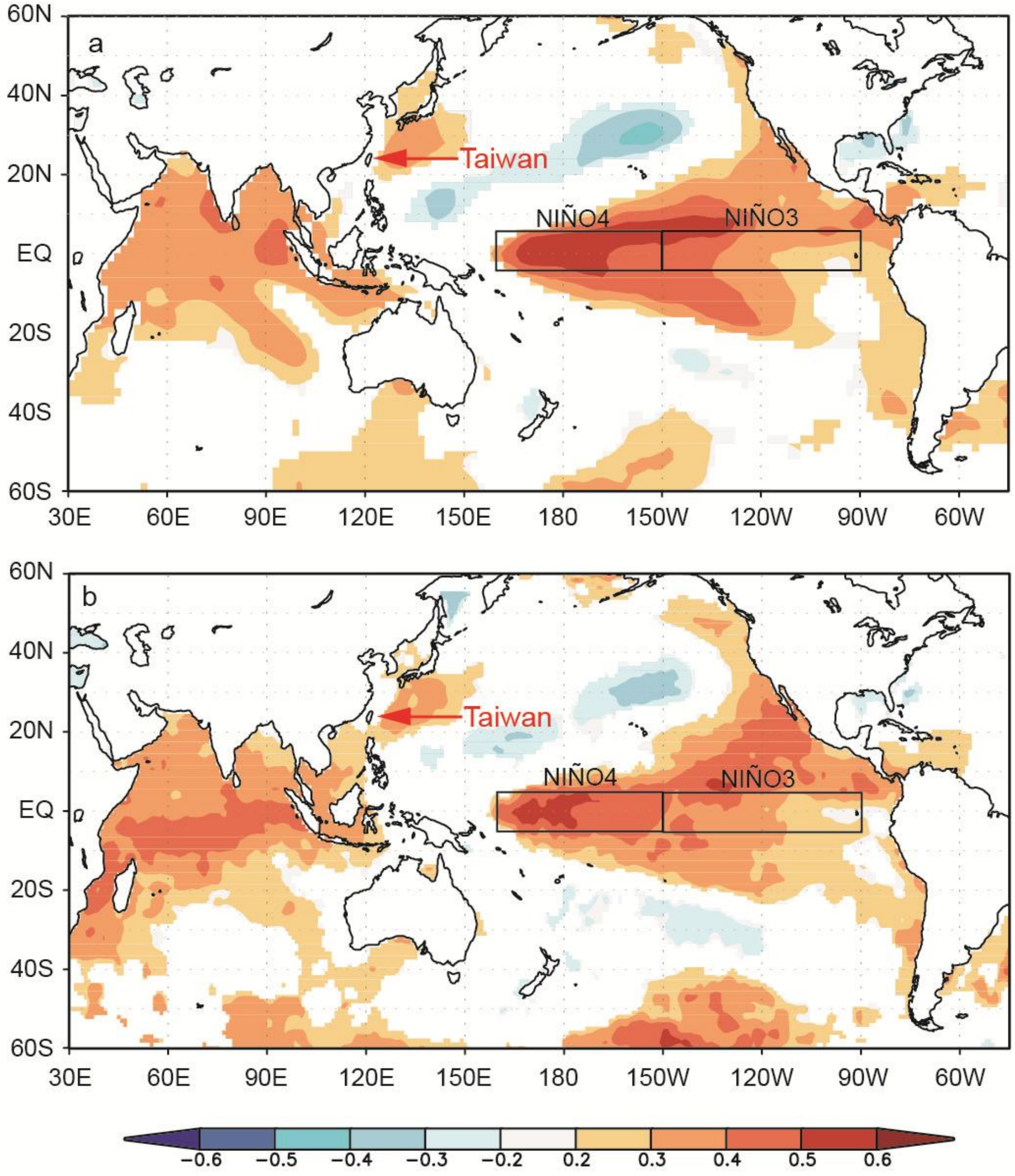

Supplementary Figure 3 | The spatial correlation field of Taiwan tree-ring δ^{18}O-based NIÑO SSTs with global SST anomaly from 1900 to 2007. a, With ERSST v3b2 [9]. **b,** with HadISST1 [10]. Colors define areas of statistically significant correlations (p<0.05). The black rectangles denote the NIÑO3 and NIÑO4 regions. This map was created by http://climexp.knmi.nl/corfield.cgi.

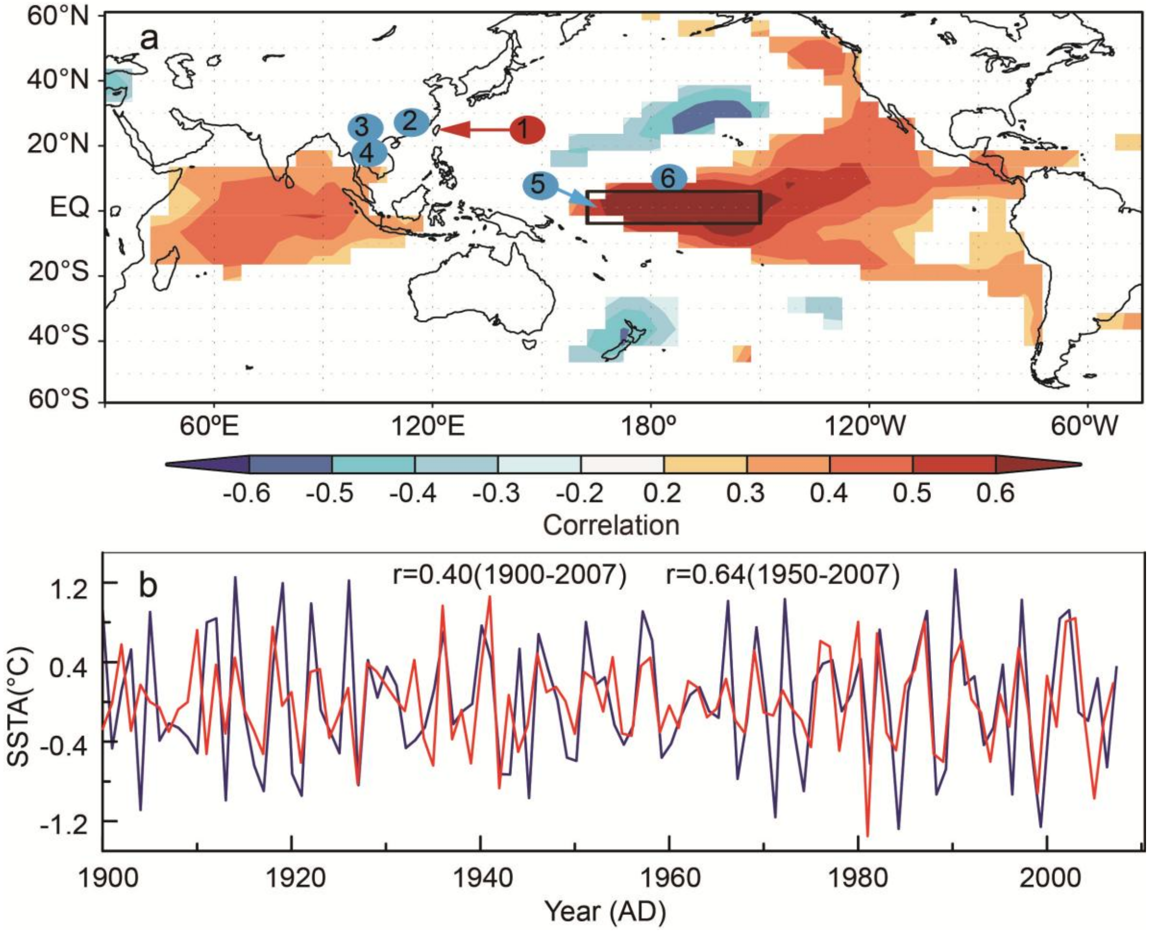

Supplementary Figure 4 | The spatial correlation field of Taiwan tree-ring δ^{18}O-based NIÑO4 SST with global SST anomaly from 1950 to 2007. a, Regression of the composite Taiwan tree-ring δ^{18}O record with global SST [11] from 1950 to 2007 AD. Colors define areas of statistically significant correlation ($p<0.05$). **b**, First order difference between the estimation based on Taiwan tree ring δ^{18}O record (red) and the NIÑO4 SST instrumental observation [11] averaged from March to May (blue) of each year, indicating that Taiwan tree ring δ^{18}O record captured the high-frequency variations of NIÑO4 SST very well. Map of Supplementary Figure 4a was created by http://climexp.knmi.nl/corfield.cgi.

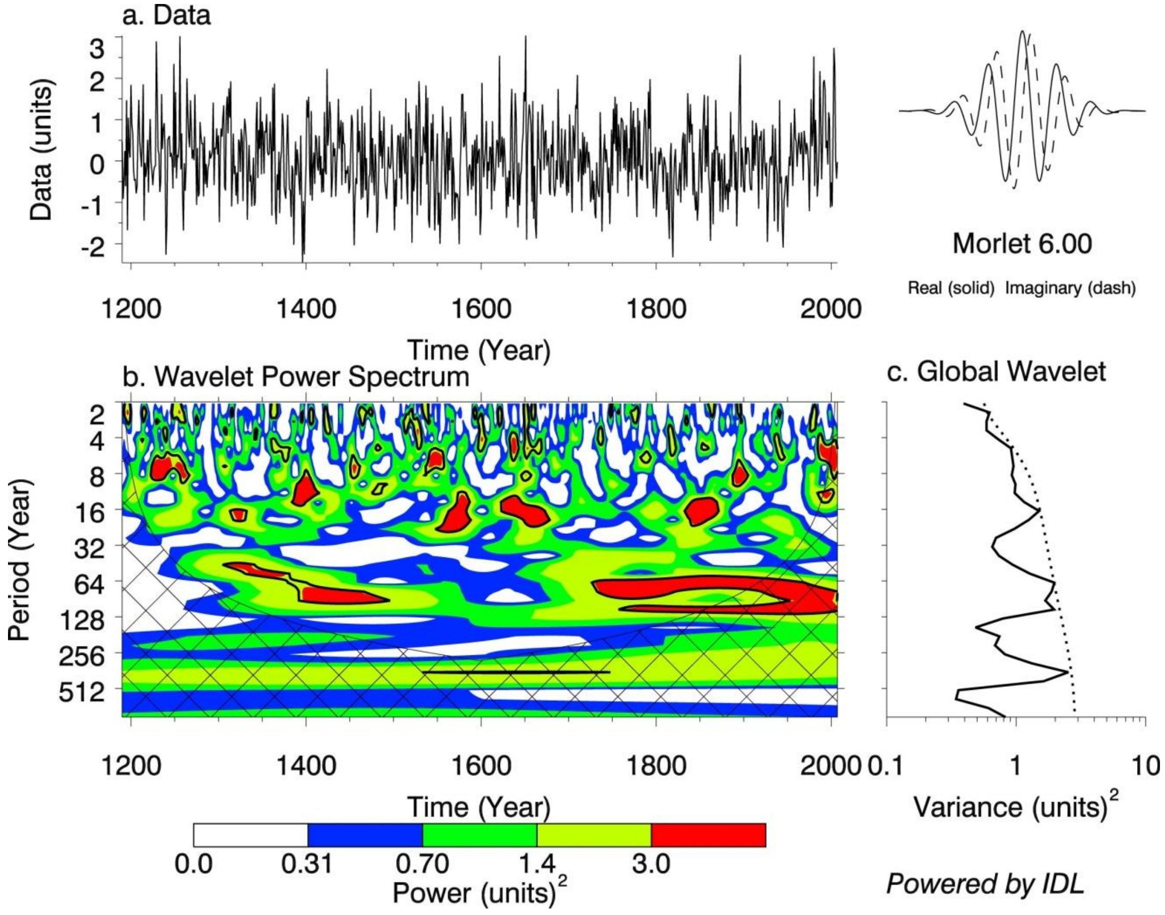

Supplementary Figure 5 | Wavelet analyses of the Taiwan tree δ^{18}O-based NIÑO4 SST reconstruction. The result of the wavelet analysis [12] shows that the interannual and decadal variabilities have persisted throughout the 818-year-long timeseries. Centennial-scale variability is strongest in the 1390–1510 and 1740–2007 periods. This map was created by http://ion.exelisvis.com/.

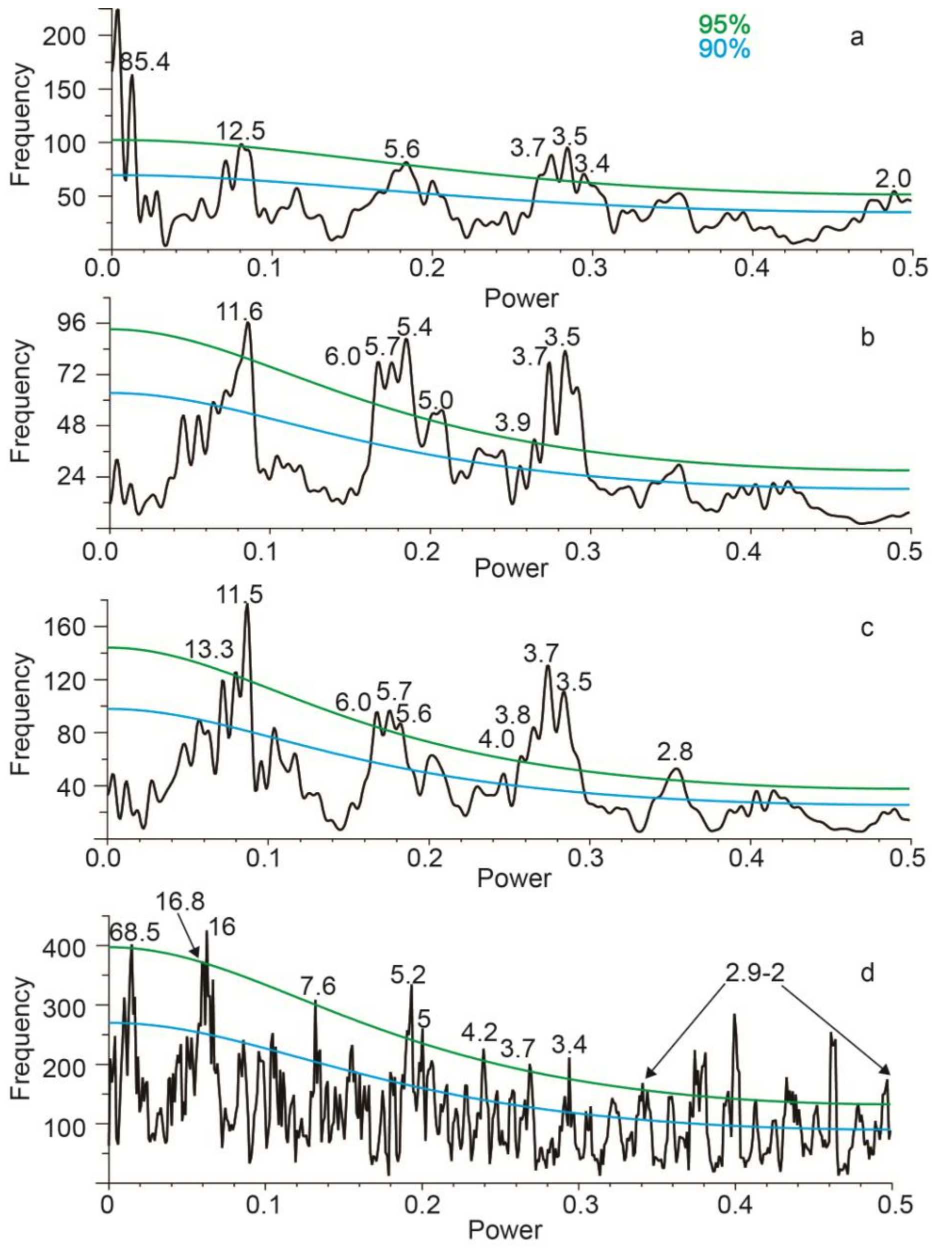

Supplementary Figure 6 | Dominant spectral features of observed and reconstructed March to May NIÑO4 SST indices. Multi-taper spectral estimates of **a,** ERSST [13] (1855–2007 AD). **b**, Hadley Centre [14] (1871–2007 AD) . **c**, Kaplan [15] (1857–2007 AD), and **d**, the 818-yr-long Taiwan δ^{18}O-based NIÑO4 SST reconstruction. There are significant interannual (2–8-year), decadal (~16-year), multidecadal (~70-year) and centennial (~100-year) periodicities in our reconstruction.

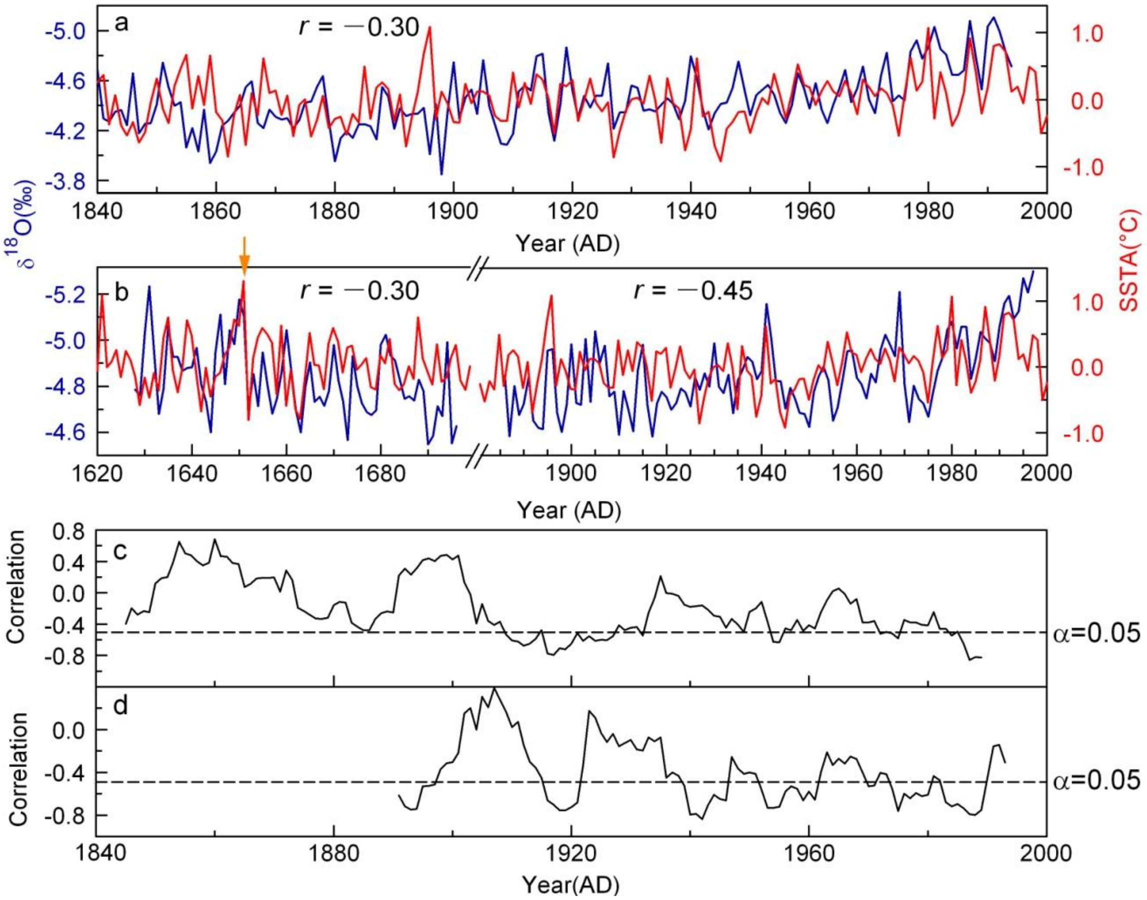

Supplementary Figure 7 | Comparison of our NIÑO4 SST anomaly reconstruction (red) with other records (blue). a, Maiana coral $\delta^{18}O$ series [16] during 1841–1994 AD. **b**, Palmyra coral $\delta^{18}O$ series [17] for discontinuous segments from 1630–1998. The arrow indicates the inferred 1651 AD very strong El Niño event. **c**, The 10-yr sliding correlations for **a**. **d**, The 11-yr sliding correlations for **b** during 1886 to 1998.

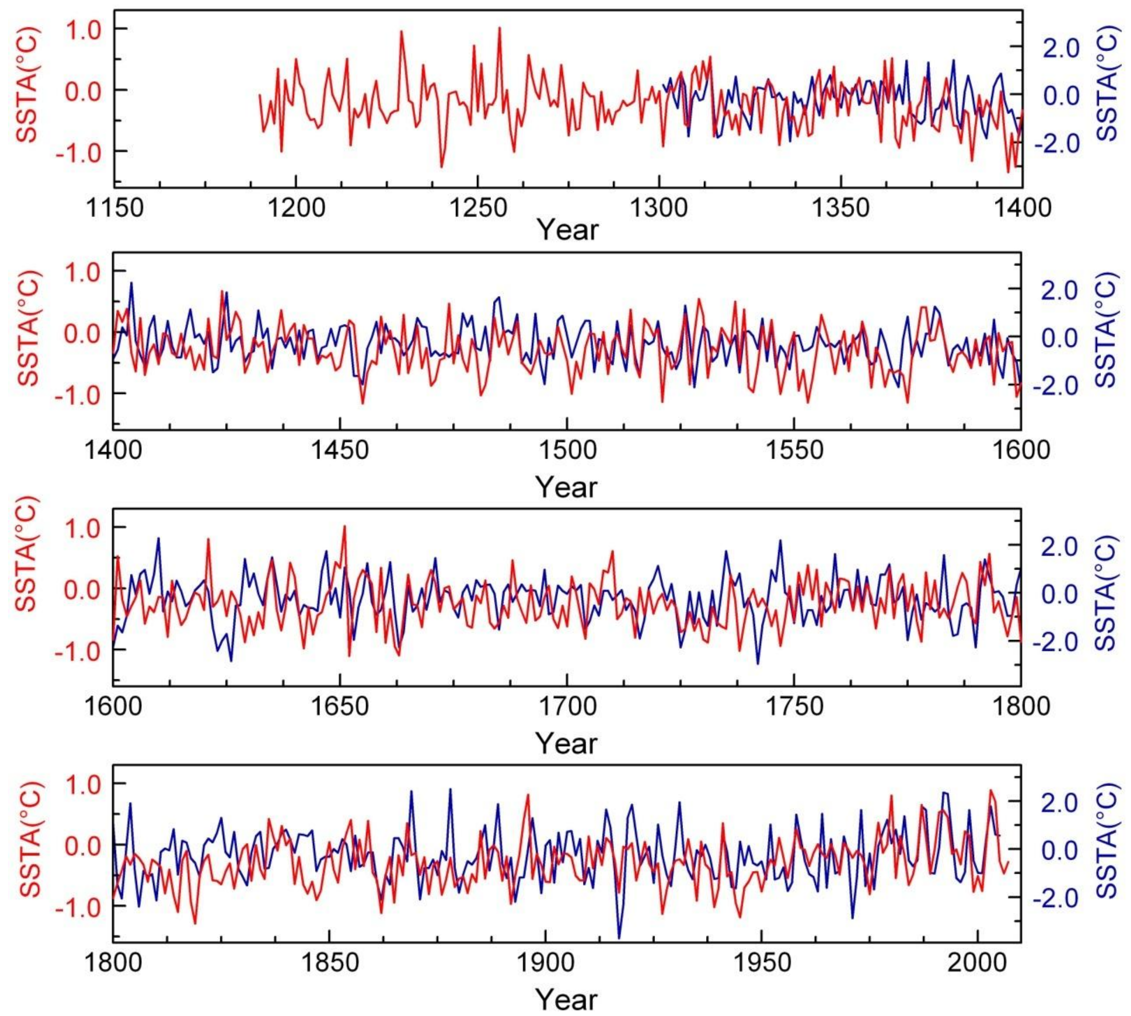

Supplementary Figure 8 | Comparison of our NIÑO4 SST anomaly reconstruction (red) with the ring-width based NIÑO3.4 SST (blue, prior November to current January) reconstruction from both the tropics and mid-latitudes spanning the past seven centuries [18].

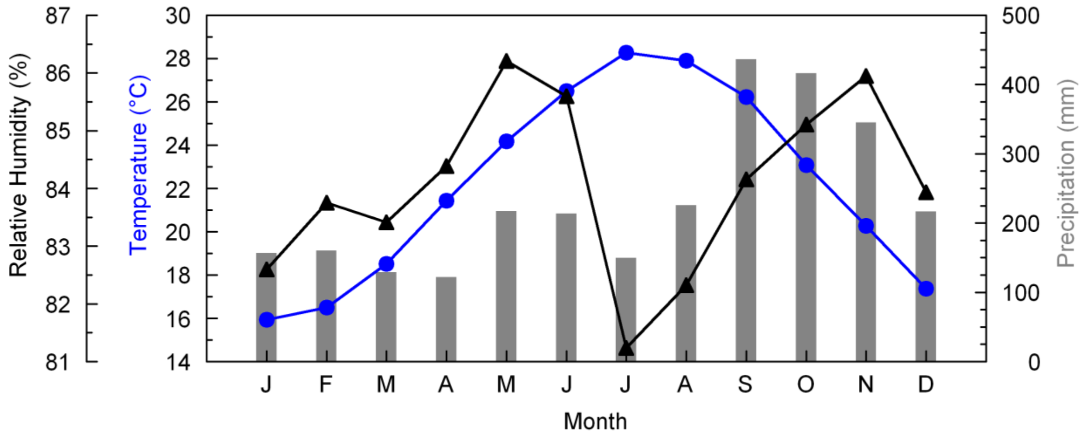

Supplementary Figure 9 | The monthly mean (1936–2007) temperature (blue), mean precipitation (gray) and mean relative humidity (black) of Yilan (24°46′N, 121°45′E, 8 m a.s.l.) station. It is located roughly 70 km from the sampling site.

Supplementary Table 1 | Statistical characteristics of (Taiwan) STD ring-width chronology *

Statistic	Standard ring-width chronology
Sample size (cores/trees)	50 /29
Average mean sensitivity	0.23
Standard deviation	0.43
Skewness	0.28
Kurtosis	0.47
Variance in PC1 (%)	27.11
First order autocorrelation (AR1)	0.59
Mean correlation among all series	0.24
Mean correlation between trees	0.24
Expressed population signal (EPS)	0.90

*The mean sensitivity is a measure of relative difference in widths between adjacent rings.

The first order auto-correlation is used to indicate that the tree-ring growth in one year is to some degree influenced by its growth in the preceding year.

The mean correlation between tree-ring series is a useful criterion, which is independent of the sample size and is an indication of the common variance [19]. The expressed population signal (EPS) is used to quantitatively evaluate the reliability of tree-ring chronologies. In general, an EPS value greater than 0.85 is considered acceptable [19, 20].

Supplementary Table 2 | Correlations within all these $\delta^{18}O$ series from different cores (*p*<0.001). All the correlation coefficients are very high, which means all the tree-ring $\delta^{18}O$ series have coherent variations. The correlation coefficients in top-right part of table are the same to those in the left-down, thus we leave them blanks. The blanks in the left-down mean the cores simply have no overlap.

r/ the effective number of degree of freedom (EDOF[21]) estimating (*in italics*).

	26B	25A	06A	X1	12B	23B	28A	04B	20A	21B	22A	01A	30B	32A	05A	18B
26B	1															
25A	0.84 /*293*	1														
06A	0.63 /*51*	0.76 /*55*	1													
X1	0.82 /*76*	0.76 /*78*	0.57 /*57*	1												
12B	0.77 /*161*	0.73 /*162*	0.51 /*49*	0.73 /*74*	1											
23B	0.81 /*258*	0.83 /*251*	0.89 /*11*	0.65 /*34*	0.84 /*140*	1										
28A	0.58 /*266*	0.59 /*262*	0.61 /*11*	0.69 /*32*	0.65 /*140*	0.63 /*285*	1									
04B	0.79 /*300*	0.76 /*296*	0.56 /*56*	0.69 /*80*	0.79 /*164*	0.85 /*277*	0.66 /*285*	1								
20A	0.76 /*165*	0.73 /*161*			0.85 /*40*	0.75 /*176*	0.62 /*194*	0.76 /*227*	1							
21B	0.81 /*169*	0.77 /*165*			0.79 /*42*	0.78 /*185*	0.62 /*197*	0.82 /*242*	0.76 /*279*	1						
22A								0.87 /*51*	0.88 /*90*	0.88 /*96*	1					
01A									0.76 /*37*	0.82 /*41*	0.84 /*93*	1				
30B											0.72 /*50*	0.72 /*184*	1			
32A												0.74 /*111*	0.75 /*112*	1		
05A											0.78 /*48*	0.83 /*181*	0.74 /*184*	0.74 /*132*	1	
18B													0.76 /*54*	0.68 /*73*	0.77 /*74*	1

Supplementary Table 3 | Correlations between Taiwan tree-ring $\delta^{18}O$, Kaplan MAM SST and other NIÑO3.4 or NIÑO4 SST proxies in the early 20th century (1900–1949) *vs* the late 20th century (post–1950). The majority of correlations between Taiwan tree-ring $\delta^{18}O$ series and other NIÑO SST proxies were slightly higher than those for the tree-ring $\delta^{18}O$ with Kaplan MAM SST (r=0.34, p<0.014) during 1900–1949. Our analyses suggest that the quality of instrumental data during the early 20th century may be somewhat reduced, as evidenced by generally higher correlations with other Pacific paleo-proxy datasets during the early 20th century (MEI is Multivariate ENSO Index).

	1900–1949	1950–
Kaplan MAM SST	0.34, p<0.014	0.73, p<0.0001, 1950–2007
Wilson, *et al*., 2010 –Corals only, (NIÑO3.4) [22]	0.45, p<0.022	0.67, p<0.0001, 1950–1998
Wilson, *et al*., 2010 –Teleconnected, (NIÑO3.4) [22]	0.40, p<0.001	0.51, p<0.0001, 1950–1998
Xu, *et al*., 2013a –Tree ring $\delta^{18}O$ only (NIÑO3.4) [3]	0.46, p<0.001	0.59, p<0.001, 1950–2007
Xu, *et al*., 2013b –Tree ring $\delta^{18}O$ only (NIÑO3.4+MEI) [4]	0.39, p<0.025	0.62, p<0.001, 1950–2002
Urban, *et al*., 2000 –Corals only (NIÑO3.4) [16], Mar to Jun SST	–0.36, p<0.03	–0.54, p<0.001, 1950–1993
Cobb, *et al*., 2003 Coral $\delta^{18}O$ (NIÑO3.4+4) [17]	–0.17, p<0.247	–0.54, p<0.001, 1950–1998
Cobb, *et al*., 2001 Sr/Ca (NIÑO3.4+4) [23]	0.20, p<0.170	0.25, p<0.083, 1950–1994
Sano, *et al*., 2012 –Tree ring $\delta^{18}O$ only (MEI) [5]	0.42, p<0.003	0.61, p<0.001, 1950–2004
Guilderson and Schrag, 1999 –Corals only (NIÑO4) [24]	–0.23, p<0.110	–0.52, p<0.001, 1950–1995
Cole, *et al*., 1993 Coral $\delta^{18}O$ (NIÑO4) [25]	–0.18, p<0.220	–0.39, p<0.013, 1950–1989
Cook, *et al*., 2008 –Tree ring width only (NIÑO4) [26]	0.07, p<0.645	0.65, p<0.001, 1950–2006

Supplementary Table 4 | Correlations between Taiwan tree-ring $\delta^{18}O$ and March to May NIÑO4 (including the 1st order difference), NIÑO3.4 and NIÑO3 SST indices from different gridded SST products, and El Niño Modoki Index [27] during the period from March to May, for the time periods indicated, and EDOF [21] (*in italics*).

All correlations are significant at the *p*<0.001 level.

		Entire series	1900–2007AD	1920–2007AD	1950–2007AD
**NIÑO4**	Kaplan	0.46 (1856–2007 AD)/*128.7*	0.58/*90.4*	0.64/*71.1*	0.73/*49.6*
	ERSST	0.45(1854–2007 AD)/*135.5*	0.52/*91*	0.59/*71.8*	0.68/*49.5*
	Hadley Centre	0.46 (1870–2007 AD)/*114.8*	0.53/*91*	0.58/*72.2*	0.71/*49*
**NIÑO4 （1st order difference）**	Kaplan	0.46 (1856–2007 AD)	0.40	0.60	0.64
	ERSST	0.45(1854–2007 AD)	0.38	0.52	0.58
	Hadley Centre	0.46 (1870–2007 AD)	0.44	0.58	0.59
**NIÑO3.4**	Kaplan	0.35 (1856–2007 AD)/*131.9*	0.43/*93.5*	0.47/*77.4*	0.61/*54*
	ERSST	0.41(1854–2007 AD)/*130.9*	0.48/*91.1*	0.54/*75.6*	0.61/*51.7*
	Hadley Centre	0.41 (1870–2007 AD)/*122.2*	0.44/*96.6*	0.49/*77.3*	0.61/*52.5*
**NIÑO3**	Kaplan	0.27 (1856–2007 AD)/*135.1*	0.31/*98.2*	0.30/*81.7*	0.41/*58.4*
	ERSST	0.33(1854–2007 AD)/*134.7*	0.38/*95.9*	0.41/*80.5*	0.44/*55.1*
	Hadley Centre	0.36(1870–2007 AD)/*125.3*	0.35/*100.6*	0.36/*80.9*	0.44/*55.5*
**EL NIÑO Modoki Index**		0.29(1870–2007 AD)/*116.3*	0.33/*90.8*	0.39/*72*	0.49/*49*

Supplementary Table 5 | Results of Split Calibration-Verification analysis for the Taiwan tree $\delta^{18}O$ data and the NIÑO4 SST from Kaplan et al., 1998 [11, 15].

Calibration					Verification						
Period	*r*	R^2	*ST*	*t*	Period	*r*	R^2	*RE*	*CE*	*ST*	*t*
1950-1979	0.72[a]	0.52	25[b]	3.67	1980-2007	0.74[a]	0.54	0.54	0.43	21[c]	6.67
1978-2007	0.74[a]	0.54	22[b]	6.47	1950-1977	0.72[a]	0.51	0.55	0.37	19[d]	3.67
1950-2007	0.73[a]	0.54	48[b]	6.88							

a, b, c, d indicates the 99.99%, 99%, 95%, 90% confidence level, respectively

Supplementary Table 6 | *F*–test [28] results between the peak variance of the late 20th century period (1988–1992 CE) and the high-variance peak in the mid-17th century (1648–1652 CE).

Statistical items	1998–1992 CE	1648–1652 CE
Mean	1.2295	1.1251
Variance	0.0090	0.0004
Numbers of Observation	5	5
Degree of Freedom	4	4
F	22.1536	
p (F<=f) one-tail	0.0054	
F_{Critical} one-tail	6.3882	

Conclusion: if $F > F_{\text{Critical}}$ (one-tail), we reject the null hypothesis (H_0: no significant difference). This is the case, 22.1536>6.3882. Therefore, we reject the null hypothesis. The variances of the two populations are different at the p=0.005 level, wherein the late 20th century is significantly higher.

Supplementary Table 7 | Correlations between our reconstructed NIÑO4 SST and other NIÑO3.4 SST reconstructions [18, 22, 26, 29]. Correlations are significant at the *p*<0.001 in all but one case. (*r* (time span) / EDOF, *p*<0.001 except the value given).

	Entire time span	Post–1700	Post–1800	Post–1900
Li, *et al*., 2013 [20]	0.21 (1301–2005 AD) / *650.3*	0.22 / *278*	0.26 / *185.7*	0.42 / *96.2*
Emile-Geay, *et al*., 2013 [29]	0.14 (1190–1995 AD) / *763.2*	0.22 / *278.9*	0.29 / *190.8*	0.39 / *98.5*
Wilson, *et al*., 2010 –Corals only [22]	0.21 (1607–1998 AD) / *341.4*	0.29 / *253.9*	0.34 / *155.6*	0.52 / *76.8*
Wilson, *et al*., 2010 –Teleconnected [22]	0.17 (1540–1998 AD) / *401.1*	0.16 (*p*<0.005) /*250.5*	0.30 / *159.5*	0.38 / *78.5*
Cook, *et al*., 2008 NIÑO4 [26]	0.17 (1300–2006 AD) / *670.3*	0.22 / *280.7*	0.28 / *186.8*	0.40 / *98*

Supplementary References:

1. Dai, A. G. Characteristics and trends in various forms of the Palmer Drought Severity Index (PDSI) during 1900–2008. *J Geophys Res.***116**, D12115, doi:10.1029/2010JD015541 (2011).

2. http://climexp.knmi.nl/select.cgi?id=someone@somewhere&field=scpdsi

3. Xu, C., Zheng, H., Nakatsuka, T., Sano, M. Oxygen isotope signatures preserved in tree ring cellulose as a proxy for April-September precipitation in Fujian, the subtropical region of southeast China. *J. Geophys. Res.***118**, 12805–12815 (2013a).

4. Xu, C., Sano, M., Nakatsuka, T. A 400-year record of hydroclimate variability and local ENSO history in northern Southeast Asia inferred from tree-ring δ^{18}O. *Palaeogeogr. Palaeoclimatol. Palaeoecol*. **386**, 588–598 (2013b).

5. Sano, M., Xu, C., Nakatsuka, T. A 300-year Vietnam hydroclimate and ENSO variability record reconstructed from tree ring δ^{18}O. *J. Geophys. Res.***117**, D12115 (2012).

6. http://climexp.knmi.nl/select.cgi?id=someone@somewhere&field=hadisst1

7. http://climexp.knmi.nl/select.cgi?id=someone@somewhere&field=cru_pre

8. http://climexp.knmi.nl/select.cgi?id =someone @somewhere&field=scpdsi

9.https://www.ncdc.noaa.gov/data-access/marineocean-data/extended-reconstructed-sea-surface-temperature-ersst-v3b

10. http://hadobs.metoffice.gov.uk/hadisst/

11. Kaplan, A., Kushnir, Y., Clement, A. C., Blumenthal, M. B., Rajagopalan, B. Analyses of global sea surface temperature 1856–1991. *J. Geophys. Res.***103**, 18567–18589 (1998).

12. Torrence, C., Compo, G. P. A practical guide to wavelet analysis. *Bulletin of the American Meteorological Society***79**, 61–78 (1998).

13. http://climexp.knmi.nl/select.cgi?id=someone@somewhere&field=ersstv4

14. http://climexp.knmi.nl/select.cgi?id=someone@somewhere&field=hadisst1

15.http://gcmd.gsfc.nasa.gov/KeywordSearch/Metadata.do?Portal=amd&KeywordPath=Parameters|CLIMATE+INDICATORS|OCEAN%2FSST+INDICES|KAPLAN+SST+INDEX&EntryId=LDEO_KAPLAN_INDICES&MetadataView=Full&MetadataType=0&lbnode=mdlb2

16. Urban, F. E., Cole, J. E., Overpeck, J. T. Influence of mean climate change on climate variability from a 155-year tropical Pacific coral record. *Nature* **407**, 989–993 (2000).

17. Cobb, K. M., Charles, C. D., Edwards, R. L., Cheng, H., Kastner, M. El Niño/Southern Oscillation and tropical Pacific climate during the last millennium. *Nature* **424**, 271–276 (2003).

18. Li, J. *et al*. El Niño modulations over the past seven centuries. *Nat. Clim. Change* **3**, 822–826 (2013).

19. Cook, E., Kairiukstis, T. L. Methods of Dendrochronology. *Kluwer, Dordrecht,* pp.1–408 (1990).

20. Wigley, T., Briffa, K., Jones, P. On the average value of correlated time series, with applications in dendroclimatology and hydrometeorology. *J. Clim. Appl. Meteorol.* **23,** 201–213(1984).

21. Bretherton, C. S. *et al.* The effective number of spatial degrees of freedom of a time-varying field. *J. Clim.* **12**, 1990–2009 (1999).

22. Wilson, R. *et al*. Reconstructing ENSO: the influence of method, proxy data, climate forcing and teleconnections. *J. Quaternary Sci.* **25**, 62–78 (2009).

23. Cobb, K. M., Charles, C. D., Hunter, D. E. A central tropical Pacific coral demonstrates Pacific, Indian, and Atlantic decadal climate connections. *Geophys. Res. Lett.* **28**, 2209 (2001).

24. Guilderson, T. P., Schrag, D. P. Reliability of coral isotope records from the western Pacific warm pool: A comparison using age-optimized records. *Paleoceanography* **14**, 457–464 (1999).

25. Cole, J. E., Fairbanks, R. G. Shen, G. T. The spectrum of recent variability in the Southern Oscillation: Results from a Tarawa Atoll coral. *Science* **260**, 1790–1793 (1993).

26. Cook, E. R., D'Arrigo, R. D., Anchukaitis, K. J. ENSO reconstructions from long tree-ring chronologies: unifying the differences? Talk presented at a special workshop on Reconciling ENSO Chronologies for the Past 500 Years, Moorea, French Polynesia, 2–3 April, 2008.

27. http://www.jamstec.go.jp/frcgc/research/d1/iod/DATA/emi.monthly.txt

28. Kong, Y. S., Qian, J. M., Zang, Z. L. Principles and Methods of Statistical Weather Forecasting. *China Meteorological Press*, *Beijing* (in Chinese) , pp.1–565 (2010).

29. Emile-Geay, J., Cobb, K. M., Mann, M. E., Wittenberg, A. Estimating central equatorial Pacific SST variability over the past millennium. Part II: Reconstructions and implications. *J. Clim.***26**, 2329–2352 (2013).
